# Multiple Transcriptome Data Analysis Reveals Biologically Relevant Atopic Dermatitis Signature Genes and Pathways

**DOI:** 10.1371/journal.pone.0144316

**Published:** 2015-12-30

**Authors:** Debajyoti Ghosh, Lili Ding, Umasundari Sivaprasad, Esmond Geh, Jocelyn Biagini Myers, Jonathan A. Bernstein, Gurjit K Khurana Hershey, Tesfaye B. Mersha

**Affiliations:** 1 Division of Immunology, Allergy & Rheumatology, Department of Internal Medicine, University of Cincinnati, Cincinnati, United States of America; 2 Division of Biostatistics and Epidemiology, Cincinnati Children’s Hospital Medical Center, Department of Pediatrics, University of Cincinnati, Cincinnati, United States of America; 3 Division of Asthma Research, Cincinnati Children’s Hospital Medical Center, Department of Pediatrics, University of Cincinnati, Cincinnati, United States of America; CNRS-University of Toulouse, FRANCE

## Abstract

Several studies have identified genes that are differentially expressed in atopic dermatitis (AD) compared to normal skin. However, there is also considerable variation in the list of differentially expressed genes (DEGs) reported by different groups and the exact cause of AD is still not fully understood. Using a rank-based approach, we analyzed gene expression data from five different microarray studies, comprising a total of 127 samples and more than 250,000 transcripts. A total of 89 AD gene expression signatures ‘89ADGES’, including *FLG* gene, were identified to show *dysregulation* consistently across these studies. Using a Support Vector Machine, we showed that the ‘89ADGES’ discriminates AD from normal skin with 98% predictive accuracy. Functional annotation of these genes implicated their roles in immune responses (e.g., betadefensin, microseminoprotein), keratinocyte differentiation/epidermal development (e.g., *FLG*, *CORIN*, *AQP*, *LOR*, *KRT16*), inflammation (e.g., *IL37*, *IL27RA*, *CCL18*) and lipid metabolism (e.g., *AKR1B10*, *FAD7*, *FAR2*). Subsequently, we validated a subset of signature genes using quantitative PCR in a mouse model. Using a bioinformatic approach, we identified keratinocyte pathway over-represented (P = <0.0006) among the 89 signature genes. Keratinocytes are known to play a major role in barrier function due to their location in the epidermis. Our result suggests that besides immune- mediated pathway, skin barrier pathways such as the keratinocyte differentiation pathway play a key role in AD pathogenesis. A better understanding of the role of keratinocytes in AD will be important for developing novel “barrier therapy” for this disease.

## Introduction

Atopic dermatitis (AD; MIM603165) is a common chronic inflammatory skin disease characterized by epidermal barrier dysfunction and immunological alterations[1]. Its prevalence has doubled in industrialized countries during the past decades. It is estimated that 15 to 30% of children and 2 to 10% of adults are affected by AD [2]. The yearly cost for treating AD has been estimated to be $3.8 billion in the US [3]. However, presently no specific or targeted therapy for AD is in clinical use [4]. Current therapeutic strategies are largely based on allergen avoidance, application of moisturizers (emollients) and topical/systemic corticosteroids or immune-suppressants, which often have significant toxicity and transient efficacy [5]. Several microarray-based study designs are routinely employed to investigate the pathogenesis of AD, including analysis of differentially expressed genes (DEGs) between AD patients and healthy controls. However, there is considerable variation in the list of DEGs reported by different groups, which might be resulting from analytic bias in which a fixed cutoff position is applied across a dataset and only genes meeting this criteria are compared. However, setting a uniform threshold (e.g. p-value cutoff) may not be very useful, since sample sizes can vary across datasets [6].

In the present study, we used an analysis approach based on biological relevance, consistency/reproducibility and statistical significance, consisting of the following steps (a) identifying DEGs from individual studies based on biological relevance (i.e. fold change, FC) [7]; (b) finding overlap between ranked gene lists across datasets using Common Dataset Ratios (CDR); (c) ranking of overlapping DEGs based on p-values; (d) using AD signature genes to discriminate AD from control samples and finally; (e) quantitative RT-PCR based validation analysis for a subset of DEGs in a mouse model of AD. This approach helps to answer several questions including: How often do the same sets of genes or pathways associated with AD occur across different datasets? and; to what degree are AD subjects from different datasets enriched for common sets of relevant genes? Answering these questions systematically in an unbiased way would allow us to better understand the pathobiology of AD and to implement better and more specific intervention strategies for this disease. Analyzing publicly accessible multiple gene expression data could be a very powerful, yet economical approach for finding disease-related genes and pathways [8, 9].

## Materials and Methods

### Data analysis strategy

**Fig 1:**
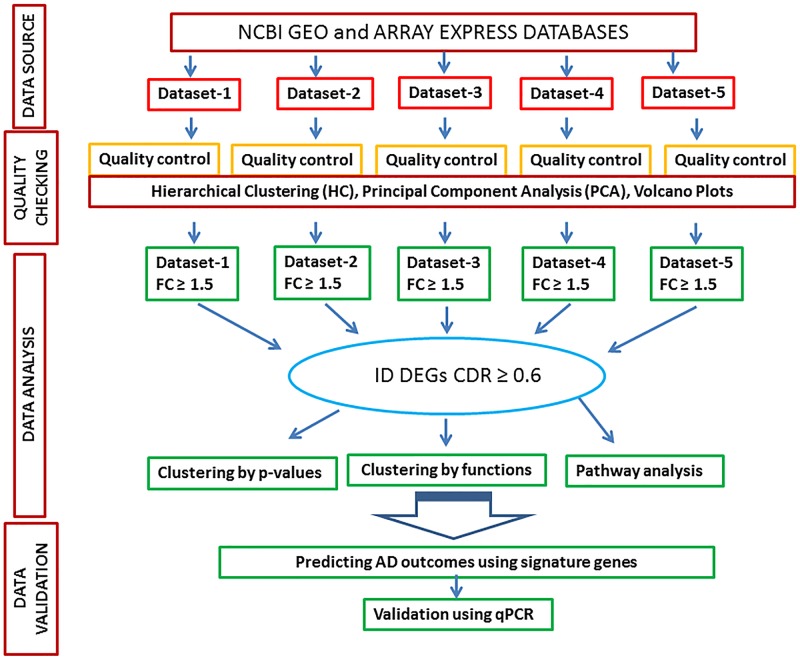
Major steps in the analysis of transcriptome data. Five individual datasets, obtained from GEO, were first normalized and quality-checked using hierarchical cluster analysis (HCA) and principal component analysis (PCA), and differentially expressed genes (DEGs; fold change ≥ 1.5) were identified from each dataset. Genes differentially regulated between AD and non-AD controls in at least 3 out of 5 datasets (common dataset ratio ≥ 0.6), were ranked by fold-change. Ingenuity Pathway Analysis (IPA) were conducted for network analysis and ACUMENTA for pathway analysis, and Support Vector Machine and discriminant analysis were used to discriminate and predict membership of AD patients from healthy controls.

To explore genes and pathways related to AD, gene expression analysis was done using AD datasets originated from five independent studies [4, 10–13]. First, we analyzed individual datasets obtained from publicly accessible databases to determine DEGs with fold changes ≥1.5 [6]. The DEGs consistently present in individual datasets were identified and arranged based on p values [6]. Our DEGs were then clustered by their functions to identify the most affected pathways related to AD. Data collection and analysis steps have been summarized in Fig 1.

### Identification of AD gene expression datasets and filtering criteria

To identify AD microarray datasets, we used the NCBI GEO (Gene Expression Omnibus, NCBI) database [14, 15]. Relevant literature examined for DEGs in AD *versus* non-AD subjects published between 2000 and February 2014 were selected using the search criteria human [organism] AND Atopic Dermatitis AND 2000/01/01:2014/02/28 [Publication Date]. Eligible datasets were selected based on the following inclusion criteria: a) the dataset must compare AD patients to healthy (non-AD) controls, and b) the dataset must be generated from same tissue type (i.e. skin). The following information was extracted from each study: (1) GEO accession numbers, (2) sample type, (3) platform, (4) numbers of AD and non-AD individuals, and (5) gene expression values. Visual BASIC macros were used to extract the expression values of individual genes in AD and control samples.

AD has been recognized as a systemic disease in the current literature [16–19]. It has a strong genetic component and often accompanied by a variety of systemic immune abnormalities. Additionally, its temporal progression to allergic rhinitis and asthma, the process known as atopic march, is a classic example for its systemic/multiorgan involvement. Therefore, we initially considered all AD samples, regardless of their patient origin (paired/un-paired) and clinical subtype (lesional/non-lesional or chronic/acute) to compare with the controls. Duplicate controls and AD samples as well as psoriasis skin data, when present in the datasets, were removed. This analysis gave us biologically relevant genes and pathways very consistant with previous individual studies (please see discussion). We then repeated the analysis using data obtained selectively from chronic lesional AD samples and control samples from the five datasets. The differential gene expression in chronic versus acute lesional stages in dataset GSE36842 has previously been described [4]. Genes differentially expressed in lesional samples compared to normals were highly consistant between the five different datasets, and were used for pathway and over-representation analysis.

### Data analysis

Human AD microarray datasets that fulfilled the inclusion criteria were downloaded from the NCBI GEO database. Five independent gene expression microarray studies, comprising a total of 127 samples and more than 250,000 transcripts representing approximately 25,000 unique genes (based on Unigene clusters) were utilized. We constructed data tables containing gene expression values, with genes/probes in rows and samples/experiments in columns using GEO2R [15], an interactive web tool that processes data tables using the GEOquery [20], and limma R packages from the Bioconductor project [21, 22]. GEOquery R package was used to parse GEO data into R data structures that can be used by other R packages. It handles a wide range of experimental designs and data types and applies multiple-testing corrections on p-values to help correct for the occurrence of false positives. We selected Benjamini and Hochberg false discovery rate (FDR) as it is the most commonly used adjustment method for microarray data and provides a good balance between discovery of statistically significant genes and false positives [23]. Transcripts present in at least 3 out of 5 datasets (CDR ≥ 0.6) were identified and sorted according to their average fold changes. Unsupervised hierarchical cluster analysis (HCA) and principal component analysis (PCA) were performed with data obtained from AD and non-AD groups using the program Genesis to detect outliers [24]. Panel A in S1 Fig shows sample hierarchical clustering, Panel B in S1 Fig principal component analysis and S2 Fig shows p-value *versus* fold-change volcano plot of GSE36842 dataset done to check the initial data quality.

### Selection of discriminatory genes

#### a) Gene signatures based on rank analysis

Transcripts with FC>1.5 and CDR ≥ 0.6 were considered for subsequent analysis. At this step, in cases where individual differentially expressed gene was associated with multiple affymetrix identifiers (Affy IDs), the ID associated with the highest fold change was considred. This gave us 89 genes/transcripts (designated as 89ADGES) consistently up/down-regulated in AD compared to controls in all datasets. These genes were used for subsequent statistical analysis, functional annotation, pathway/network and over-representation analysis. To check the statistical significance, transcripts of each dataset were sorted by p-values (low to high) and top 5% of genes were considered most significant in each dataset. For example, dataset GSE6012 contains data for a total of 22283 transcripts, so 1114 transcripts ranked above 5% significance level (22283 x 5/100 = 1114), which included both up and down-regulated genes. Using the p-value or FDR-adjusted p-value did not appreciably change the order of this arrangement. Using this approach, the highly significant DEG, *S100A7* (Affy ID: 205916_at) came at the 5^th^ position within 22283 transcripts and the percent significance level/rank was 0.022. However, this value for an individual transcript can vary between datasets depending on its position in the dataset and an average percent rank value could be determined for the 89ADGES from the five different datasets, which was plotted against average fold-change.

#### b) Classification and prediction of AD patients using discriminant and support vector machine

We identified ‘signature’ genes overlapping among the five GEO datasets and attempted to ascertain whether these ‘signature’ genes can be used to discriminate AD patients from healthy controls in the samples. There were 89 top ranked DEGs shared among the five datasets (CDR ≥ 0.6). This 89-gene signature (89 AD Gene Expression Signature, “89ADGES”) was then used for prediction. This was done by randomly choosing 10% of the subjects for testing, and the rest for training. Support vector machine (SVM) was used to analyze data [25]. For SVM, the program COMBAT was used to handle batch effect [26]. SVM represents a powerful technique for general (nonlinear) classification, regression and outlier detection, and has been widely used in many bioinformatics application. The SVM function in R package e1071 was used to build the statistical prediction model with C-classification and radial kernel, where the parameters were tuned to give the best prediction results. Using the training data, a final model, which was used to predict AD in the test dataset was obtained with 10-fold cross-validation.

#### c) Bioinformatics of signature genes

The 89 DEGs (89ADGES) along with their corresponding average fold-change values were uploaded to Ingenuity Pathway Analysis (IPA) software (http://www.ingenuity.com/products/ipa). IPA generated a network ranked score, which gives the likelihood that the set of genes in a given network could be explained by chance alone. Networks with a score >3 have >99% confidence that the genes included in the network are not generated by chance [27]. The “89ADGES” signature genes identified were then mined by Literature Lab^™^ from Acumenta Biotech for pathway analysis [28, 29]. Literature Lab is an interface between experimentally-derived gene lists and scientific literature in a curated vocabulary of 24,000 biological and biochemical terms. It employs statistical and clustering analysis on over 15.73 million PubMed abstracts (01/01/90 to the present) to identify pathways (809 pathways), diseases, compounds, cell biology and other areas of biology and biochemistry. The analysis engine compares statistically the submitted gene set to 1,000 randomly generated gene sets to identify terms that are associated with the gene set more than by chance alone (http://acumenta.com/).

#### d) Validation of ‘89ADGES’ subset in mouse model of AD

Expression profiles of a subset of the 89 DEGs were evaluated using quantitative real-time polymerase chain reaction (qRT-PCR) in a mouse model that shows all hallmarks of AD. Briefly, BALB/c mice (Harlan Laboratories, Indianapolis, USA) maintained in a specific pathogen–free environment were used for this study following ethical guidelines of the Institutional Animal Care and Use Committee approved by the Veterinary Service Department of the Cincinnati Children’s Hospital Medical Center Research foundation. Mice (AD and control; seven per group) were anesthetized, backs shaved 1 day before the first allergen exposure, and either 200 μl of saline water or *Aspergillus fumigatus* extract (Greer Laboratories, Lenoir, NC, USA) was applied using a 2×2 cm patch of sterile gauze. Details related to the AD model is described elsewhere[30]. Quantitative PCR analysis was performed using primer sequences of genes as shown in S1 Table.

## Results

### Differentially expressed genes (DEGs) and signature genes

#### Characteristics of the datasets

Table 1 contains the accession numbers of the datasets, tissue, platforms along with the numbers of samples from AD patients and healthy individuals. Our database search returned five GEO datasets with accession numbers: GSE36842, GSE32924, GSE16161, GSE5667 and GSE6012. The datasets contained a total of 127 samples of which 80 were obtained from AD skin biopsies and the rest were controls. Next, we found that 3 out of the 5 available datasets (GSE36842, GSE32924 and GSE5667) contained data obtained using patient-matched lesional and non-lesional AD skin samples, with one of them (GSE36842) further discriminating between chronic and acute stages. In case of other two studies, chronic lesional AD skin samples were used. Thus all of the five datasets contained data from chronic lesional skin biopsy samples, which was utilized to identify DEGs consistent between the five datasets in case of lesional AD.

**Table 1:** Datasets used for the present study. NCBI GEO accession numbers, sample tissue types, microarray platform, samples size and the published references associated with the datasets are shown.

Data	GEO ID	Sample type/biopsy	Platform	Sample size	References
**1**	GSE36842	Skin	Affymetrix (GPL570)	**39**	[4]
**2**	GSE32924	Skin	Affymetrix (GPL570)	**33**	[13]
**3**	GSE16161	Skin	Affymetrix (GPL570)	**18**	[10]
**4**	GSE5667	Skin	Affymetrix (GPL96)	**17**	[12]
**5**	GSE6012	Skin	Affymetrix (GPL96)	**20**	[11]

#### Characteristics of differentially expressed genes

Fig 1 shows the major steps of data analysis. A total of 250,000 transcripts from the five available independent datasets were investigated. The DEGs that exhibited the highest average fold change values were: SERPINs (*SERPINB4*; encode inhibitor proteins for serine protease enzymes), *CDSN* (*Corneodesmosin*), *BTC* (betacellulin), *c1orf68* (chromosome 1 open reading frame 68; encodes a skin-specific protein known as LEP7) and Aldo-keto reductase (*AKR1B10*; related to inflammation). DEGs related to structure (e.g. *CORIN*, *AQP*), innate immune function (e.g. betadefensin, microseminoprotein) and cytokines, chemokines and adhesion molecules (*CCL18*, *CCL22*, *CCL17*, selectin E, skin-derived TGF-beta inhibitor Gremlin 1, epiregulin) were observed. S2 Table shows up-regulated and S3 Table shows down-regulated genes with the respective average log fold-change and p-values. We found that genes showing low p-values and high expression fold changes were related to epidermal barrier function (e.g. aquaporin, loricrine, claudin, Sciellin), anti-microbial defense (microseminoprotein, defensin) and cytokines/ chemokines (*IL37*, *CCL18*).

### Multivariate statistical analysis of DEG supported the “89ADGES” signature genes

**Fig 2:**
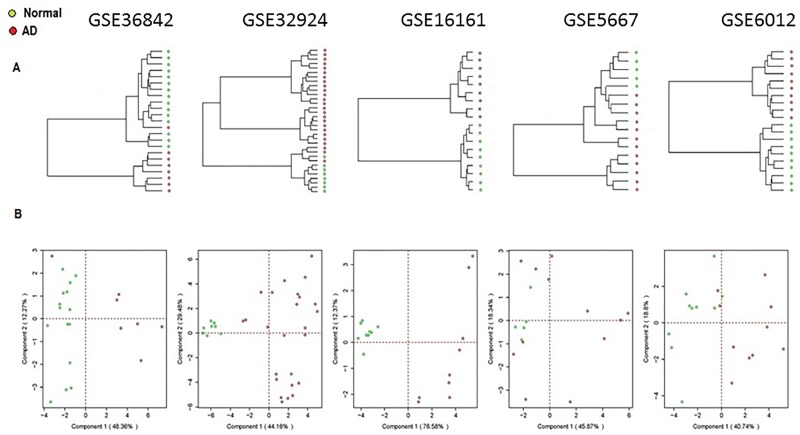
Hierarchical clustering and principal component analysis. Hierarchical cluster analysis (panel A) and principal component analysis (Panel B) are shown. HCA which is based on the means for all individuals from AD and control individuals was used to obtain similarities among individuals according to their correlation measures across all expression values of 89 gene datasets. Branch height represents dissimilarity. Note that, AD samples differ in branch length from controls. Sample-based principal component analysis (PCA) analysis of the entire AD and control samples are shown. The first PC accounted for more than double the variance of the second PC. The contribution of the first two PCs in classifying AD and control samples are presented.

**Fig 3:**
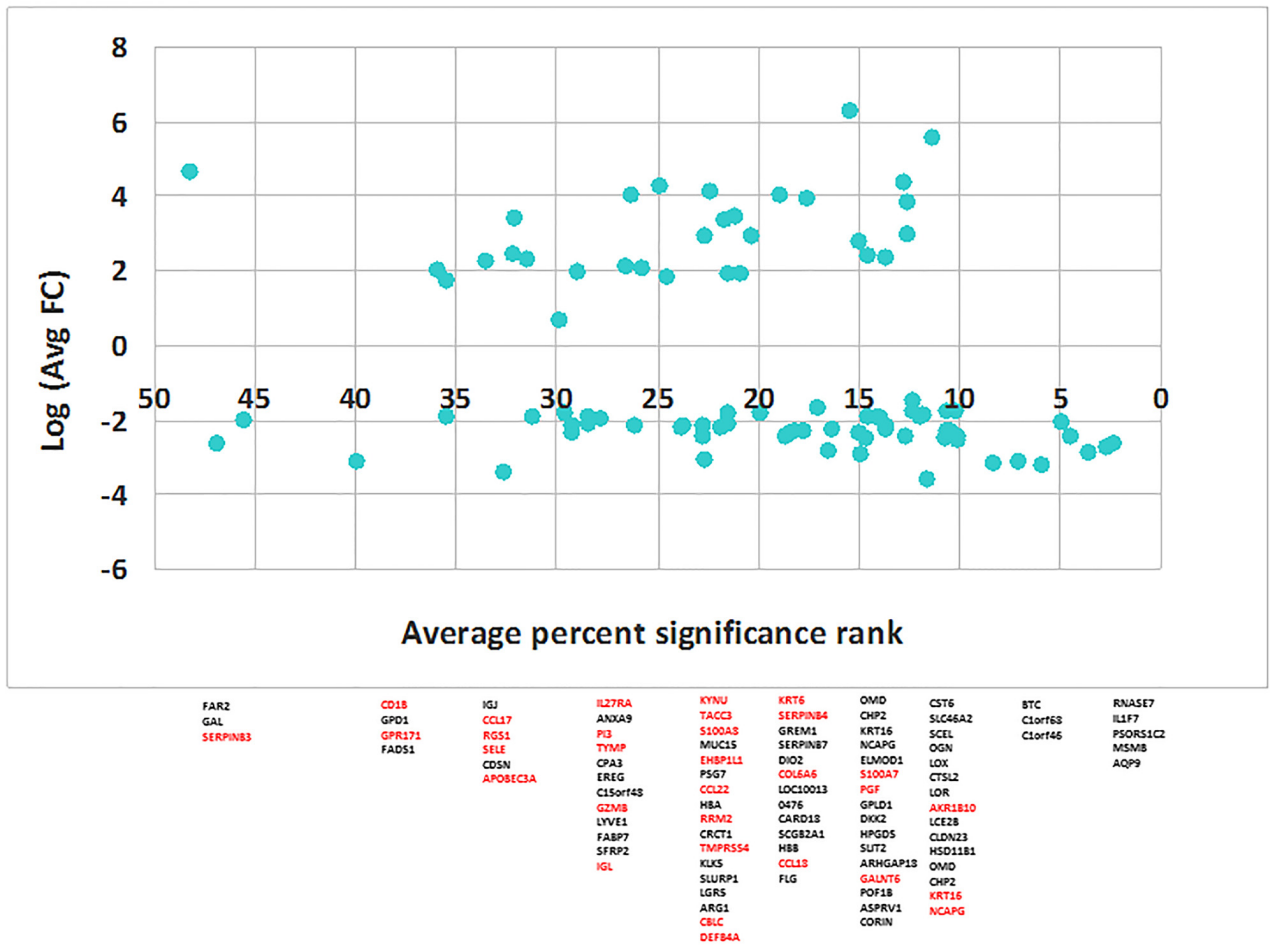
Distribution of fold change and average percent significance rank of 89ADEGS. Identification and ranking of differentially expressed genes (DEGs) from individual studies using both ranked statistical significance (x-axis) and biological relevance (fold change, FC, y-axis) provide better understanding of relevant gene sets than either of the methods. The p-values of the 89ADGES member genes were arranged (smaller to larger p-values) in each of the datasets and percent-ranked. For example, the percent-rank of a DEG/transcript at the 500^th^ position within a set of 50,000 transcript will rank 5^th^. Average percent ranks each 89ADGES member gene was determined by averaging the percent-ranks from 5 datasets and plotted against respective average fold-change values. A lower percent rank indicates higher significance (Please see S2 Table for details). Upregulated genes are in red color.

Fig 2 shows unsupervised hierarchical cluster analysis (HCA) (panel A) and Principal component analysis (Panel B) of the datasets. In HCA, euclidean distance and complete linkage were used to obtain similarities/dissimilarities among sets of individuals according to their expression values of all 89 genes. Branch height represents dissimilarity. As expected, samples were clustered into two groups: AD patients *versus* healthy individuals. Principal component analysis (PCA) of the entire AD and control samples are shown in Fig 2 (Panel B). The first PC accounted for more than double the variance of the second PC. Further analysis using principal component scatter plot showed that the first two PCs contributed to 60–80% of the total variation in AD. Fig 3 shows log average fold-changes of expression of 89DEGs plotted against p-value ranks. Result shows that the top significant genes belong to keratinocyte differentiation, cell morphology as well as some innate immune genes, which were consistently down-regulated.

### Functional annotation and enrichment analysis group the 89 DEGs into five clusters

**Fig 4:**
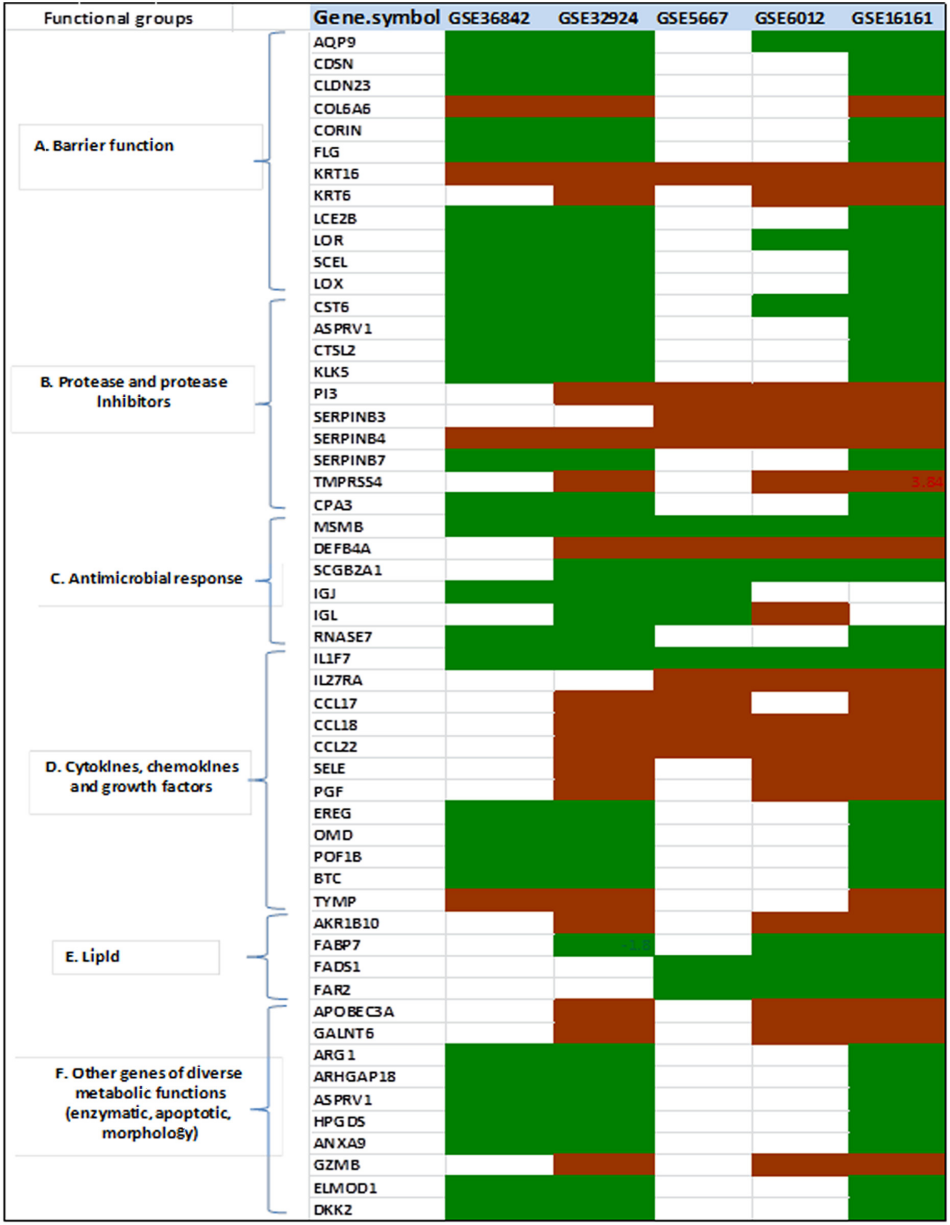
DEGs classified by functions. A, Barrier function; B, protease-protease inhibitor homeostasis; C, anti-microbial response genes; D, cytokine/ chemokine/ growth factor; E, lipid genes, and F, genes with diverse metabolic functions. Color codes: white, no expression data for that particular gene exist in the GEO dataset; red, up-regulated; green, down-regulated in AD.

Fig 4 shows the clusterning patterns of DEGs using functional categories as follows:

#### Cluster I: Epidermal development and barrier function related genes

The top structural or barrier function-related genes identified were cytokeratin (*KRT16*), collagen (*COL6A6*), late cornified envelope (*LCE2B*), loricrin (*LOR*), filaggrin (*FLG*), Sciellin (*SCEL*) and, aquaporin (*AQP9*). *KRT16* and *COL6A*6 were up-regulated in atopic dermatitis skin while the rest were down-regulated. Interestingly, a number of genes of this group were significantly associated with the keratinocyte differentiation pathway.

#### Cluster II: Cytokines/chemokines and inflammation related genes

Down-regulation of *IL1F7* expression (now known as IL37) was observed in all of the datasets. Other differentially regulated cytokine/chemokine-related genes were *SLURP*1 (secreted LY6/PLAUR domain containing 1), the chemokines *CCL17*, *CCL18 CCL22* and the growth factors *EREG* (epiregulin), *PGF2* (placental growth factor), *OGN* (osteoglycin), *SELE* (E-selectin) and thymidine phosphorylase (*TYMP*). All chemokines were up-regulated with concomitant down-regulation of growth-factors in AD. In addition, IL27 receptors were up-regulated in AD datasets.

#### Cluster III: Proteases and protease inhibitors related genes

Top differentially expressed protease and protease-inhibitor genes identified were *CORIN* (membrane-bound serine peptidase), Kallikriens (*KLK5*; trypsin-like serine protease), serpins (*SERPINB3*, *SERPINB4*, *SERPINB7*), *PI* (protease inhibitor epidermal), Cystatin (*CSTM*), *ASPRV1* (aspartic peptidase, retroviral-like 1) and Cathepsin (*CTSL2*). Among these, we found that epidermal proteases were up-regulated in AD whereas protease inhibitors were down-regulated. TMPRSS4 is a transmembrane serine protease, which was up-regulated more than 6-fold in AD samples.

#### Cluster IV: Anti-microbial response related genes

The functional cluster of protease-protease inhibitors was followed by another group of DEGs related to *anti-microbial function*. We identified pronounced down-regulation of the following genes related to innate immunity in AD datasets: *MSMB* (microseminoprotein beta), *LTF* (lactotransferrin) and *SCGB2A1* (secretoglobin 2A1). The Beta-defensin-2 encoded by the *DEFB4* (defensin, beta 4), also known as skin-antimicrobial peptide 1 (*SAP1*), was upregulated in AD compared to controls, which might represent a general response to bacterial infection [31].

#### Cluster V: Metabolic functions and pathways related genes

This cluster consisted of genes encoding epidermal enzymes that were significantly down-regulated in AD tissues. Top DEGs of this group were *AKR1B10*, *GALANT6* and *ARHGAP18* with average log fold change values of 4.9, 2.2 and -2.1, respectively. Other DEGs of this group included *DKK2* (dickkopf WNT signaling pathway inhibitor 2), *ELMOND*1 (ELMO/CED-12 domain containing 1), and *RGS1* (regulator of G-protein signaling 1), which have a wide range of regulatory roles in metabolic pathways.

#### Cluster VI: Genes related to lipid metabolism

This cluster consisted of a set of genes including *FADS1* (fatty acid desaturase 1), *FAR2* (fatty acyl CoA reductase 2), *FABP7* (fatty acid binding protein 7), and *GPD1* (glycerol-3-phosphate dehydrogenase 1) involved in epidermal lipid metabolism and homeostasis. Member genes of this cluster were downregulated in all of the datasets analyzed, clearly indicating an altered expression pattern of lipogenic genes/pathways in the AD skin.

### AD signature genes ‘89ADGES’ discriminate AD from control subject with 98% accuracy

Next, the 89ADGES were used to determine prediction accuracy. We randomly chose 30 subjects (19 out of 80 AD, 11 out of 47 normal) for testing, and the rest for training. Using the training data with 10-fold cross validation, we obtained an average accuracy of 95% (ranged from 90 to 100%). The prediction accuracy of the testing data was 98% (S4 Table).

### Network analysis revealed several genes involved in epidermal barrier, inflammation and immunity, anti-oxidant and lipid metabolism were over represented among 89ADGES more than by chance alone

**Fig 5:**
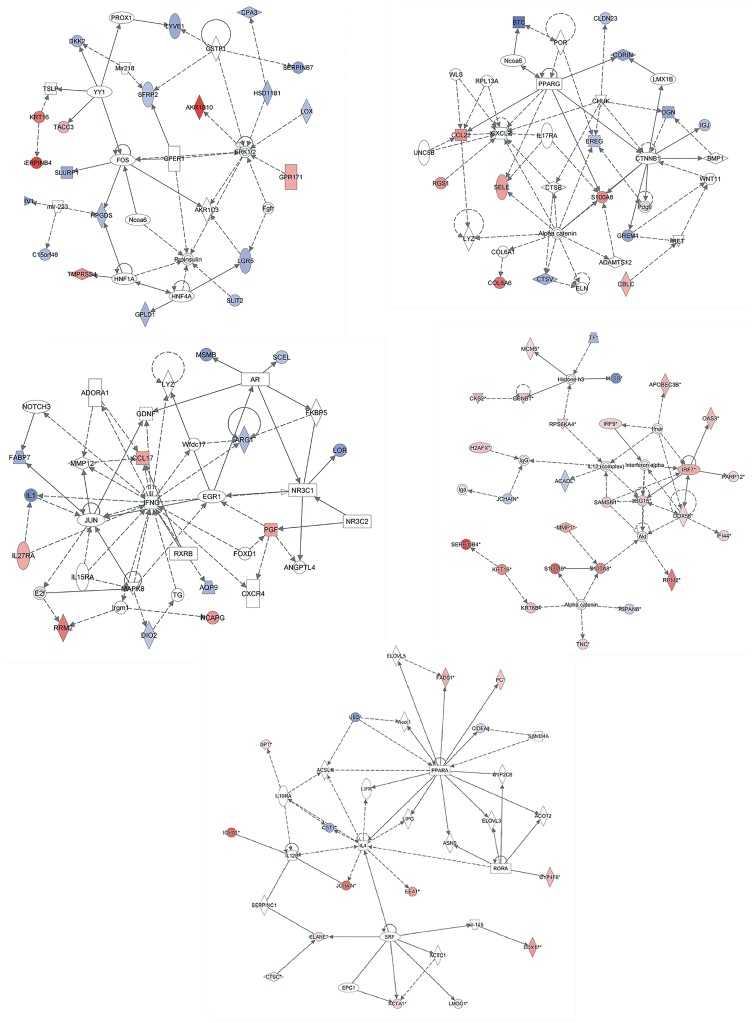
Top biological networks identified using IPA connecting several DEGs for (a) lesional and non-lesional combined (b) lesional (c) non-lesional AD. Lipid genes, protease genes and immune-related ‘nodal genes’, controlling several DEGs, are located at the center of the networks. Solid lines represent direct and established regulatory functions, while dotted lines represent indirect roles.

Analysis of 89ADGES using IPA generated three different gene networks Fig 5A: dermatological diseases and conditions, Fig 5B: cellular growth and proliferation, and Fig 5C: metabolic disease; The networks had several genes involved in epidermal barrier, inflammation and immunity, anti-oxidant and lipid metabolism (see discussionsection). Similar analysis has been done for lesional (Fig 5D) and non-lesional (Fig 5E) datasets. The biological functions of these genes were related to skin development and function, cell-mediated immune response, hematology/immune cell trafficking and organ morphology (Table 2).

**Table 2:** IPA summary of associated networks and molecular and cellular functions for the 89ADGES signature genes. IPA identified three significant gene networks using 89ADGES. Associated network functions, scores and number of member genes has been shown.

**Associated Network Functions**	**Network Score** *	**Associated key genes**
Dermatological diseases and conditions	20	*KRT16*, *LOX*, *TMPRSS4*, *SERPINB4*, *AKR1B10*, *GPR171*, *TACC3*
Cellular movement, organismal development	14	*CCL22*, *CBLC*, *CORIN*, *EREG*, *S100A8*, *SELE*, *OGN*
Inflammatory response	6	*CCL17*, *CCL22*, *S100A8*, *SELE*,*RGS1*
**Molecular and Cellular Function**	**P-value** #	
Skin development and function	8.72E-07-4.91E-02	*CTSV*,*KLK5*,*LOR*, *OGN*, *KLK5*, *ASPRV1*, *CST6*, *CTSV*, *KRT16*, *SCEL*
Cell-mediated immune response	2.47E-05-2.83E-02	*CCL17*,*CCL22*,*CTSV*,*KLK5*,*RGS1*,*SELE*, *RGS1*, *IL27RA*
Immune Cell Trafficking	2.47E-05-4.65E-02	*CCL17*,*CCL22*,*CTSV*,*KLK5*,*RGS1*,*SELE*

* Networks with score >3 have a 99.9% confidence of not being generated by random chance.

^#^ The IPA computes p-values of statistical significant findings by comparing the number of molecules of interest relative to the total number of occurrences of these molecules in all functional annotations stored in the Pathways Knowledge Base (Fisher’s exact test with p-value adjusted using Benjamini-Hochberg multiple testing correction).

IPA: Ingenuity pathway analysis.

### Pathway analysis revealed that the keratinocyte differentiation pathway was highly enriched among 89ADGES’ signature genes

**Fig 6:**
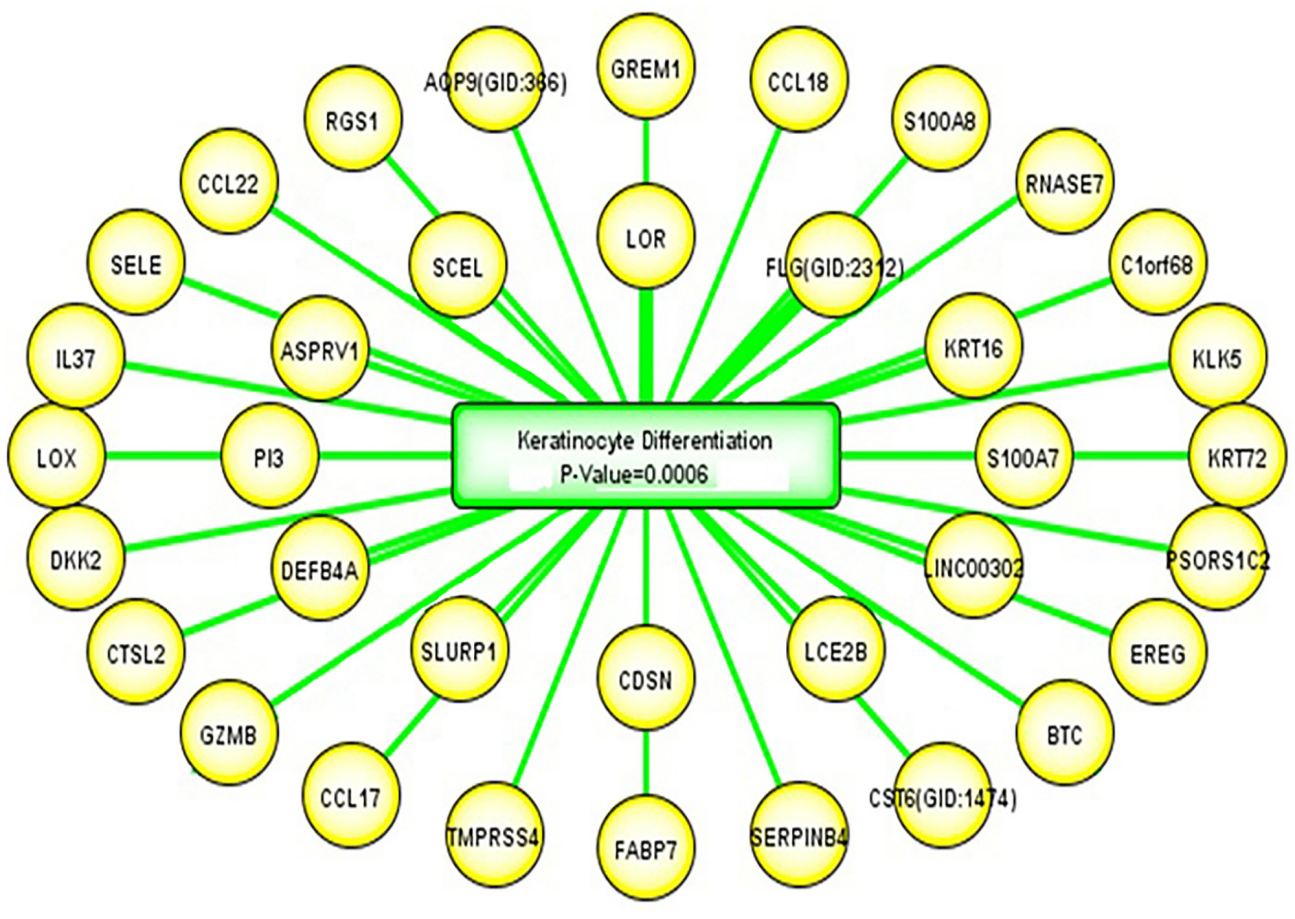
Analysis of the ‘89ADEGs’ with the ACUMENTA literature lab demonstrated an enriched of the keratinocyte differentiation pathway. Keratinocytes are known to play a major role in barrier function in AD due to their location in the epidermis. DEGs associated with the keratinocyte differentiation pathway have been shown. The biological functions of these genes were related to cellular movement, barrier functions, signaling as well as cellular development and function. The genes making the strongest contribution are shown in the inner ring starting at the 12 o’clock position and descending in a clockwise direction and outward in the order of contribution to the association to keratinocyte differentiation pathway. Thus *LOR* accounts for the largest share of the gene set's association with the keratinocyte differentiation pathway, followed by *FLG* then *KRT16* and so on. The gene in this diagram contributing the least to the gene set's association with the keratinocyte differentiation pathway is *AOP9*.

Analysis of the 89ADGES with the ACUMENTA (www.acumenta.com) revealed that the keratinocyte differentiation pathway was the most significantly enriched pathway (p = <0.0006). The genes associated with the keratinocyte differentiation pathway (36 out of the 89 signature genes) are shown in Fig 6. Many of these genes involved in barrier function [10, 32–34]. These results strongly indicate that AD is associated with a defect in the keratinocyte differentiation pathway, which is consistent with down-regulation of terminal differentiation proteins. The contributing genes for the keratinocyte pathway, ranked by the number of the corresponding publications, were *LOR* (44%), *FLG* (42%), *KRT16* (4.5%), *S100A7* (2%), *C1orf46* (1.4%), *DEFB4*A (0.6%) and *SERPINB4* (0.5%) [35].

### Validation of AD ‘signature’ genes

In order to validate our signature genes, RNA samples isolated from a mouse model of AD were used to measure gene expression in the top eight (approx. top 10%) of the signature genes. These genes (*S100A8*, *SERPIN3A*, *KRT6B*, *CLDN23*, *DEFB4*, *CCL22*, *LOR* and *CST6*) belong to the keratinocyte structure and immunologic pathway. Consistent with our human data, quantitative PCR showed a similar direction of gene-regulation in an AD mouse model (S3 Fig), which served as a technological confirmation.

### Comparison of lesional AD with healthy skin gene expression

When we compared chronic lesional AD samples from all the datasets with the control sample data, the top 10 up-regulared genes found were *SERPINB*, *AKR1B10*, *S100A9*, *LTF*, *SERPINB3*, *RRM2*, *MMP12*, *FOXM1*, *S100A8* and *CCL22*, while the top down-regulated genes included *CORIN*, *LOR* and microsenoprotein. Additionally, using a similar approach, top up-regulated genes in non-lesional AD include *IL-37*, *MSMB*, and Gremlin. The top down-regulated transcripts include keratin, beta-defensin and lipid synthesis genes (data not shown). Functional annotation categories using DEGs from lesional AD include: (a) cellular growth and proliferation (b) lipid metabolism and (c) molecular transport with the top network shown in Fig 5D. On the other hand, top functions related to DEGs identified for non-lesional AD were: (a) lipid metabolism, (b) small molecule biochemistry, (c) cell signaling, interaction and (d) cellular movement and (e) cell death and survival. The associated top network is shown in Fig 5E.

To dissect the relationship between lesional and non-lesional AD, we further compared the gene expression data derived from lesional and non-lesion skin samples included in our datasets (GSE36842, GSE32824 and GSE5667). Table 3 shows the top 10 DEGs (FC ≥ 1.5) that were consistently appeared in these datasets. Average fold-change and lowest p-values have been indicated. Our study suggests that AD-lesions were associated with up-regulation of genes encoding serpins (*SERPINB4*, *SERPINB3*) and S100 proteins (*S100A8* and *S100A9*) with beta-defensin, *AKR1B10*, *CCL18* and epidermal protease inhibitor *PI3*.

**Table 3:** Comparison between lesional and non-lesional AD. Top 10 DEGs consistently present in three of the datasets (GSE36842, GSE32924 and GSE5667) have been shown with average fold-change (lesional/non-lesional) and lowest p-values among the three datasets. Top common differentially regulated genes (according to Swindell et al[36]) in Psoriasis plaques have been indicated by asterisk (*).

Top 10 up-regulated genes		Top 10 down-regulated genes	
Gene Name	Ave. FC	Gene Name	Ave. FC
SERPINB4*	7.72	LEP	0.36
S100A9*	6.67	CYP4F8	0.42
SERPINB3*	6.51	FABP7*	0.44
MMP12*	5.91	FADS1	0.46
MMP1	5.21	TF	0.48
DEFB4A*	4.66	ZBTB16	0.50
AKR1B10*	4.65	GYG2	0.52
CCL18*	4.62	ACADL	0.54
PI3*	4.53	SORBS1	0.55
S100A7*	4.52	LONP2	0.63

In addition, we also checked the overlap between 89ADGES and differnetially expressed genes identified for Psoriasis (PS). Analysis of publicly available psoriasis transcriptome data generated by independent research groups (GSE13355, GSE14905, GSE30999) has been performed and differential gene expression in a total of 163 PS lesions versus uninvolved skin has been described by Swindell et al. [36]. Top 35 up-regulated and 35 down-regulated genes provided by Swindell et al were compared with 89ADGES. Shared genes (upregulated and down-regulated) are indicated by asterisk (Table 3). Our similarity analysis identified DEGs that were highly upregulated in PS (e.g. *ATP12A*, *RGHH*) but not significantly changed in AD. Exploring genetic overlap between AD and PS can help identify important common biological pathways that could be relevant for identifying therapeutic targets for both diseases [37].

## Discussion

Gene expression signatures are commonly used as biomarkers to classify patients from healthy controls. As an initial analysis, we considered all AD samples, regardless of their clinical subtype (lesional/non-lesional), and compared the ‘AD’ with the ‘control’ group. Next we identified lesional and non-lesional AD subtypes from each dataset and compared each subtype to the control samples. DEG and associated functional categories such as cell differentiatiation, lipid metabolism, and microbial defense defence identified using combined samples were also identified by using lesional or nonlesional subgroup analysis. However, the top network in the non-lesional group showed a clear Th2 bias, with *IL-4* as one of the major ‘nodal’ genes, while in the non-lesional subgroup, a mixed Th1-Th2 response seemed to drive the clinical manifestation of the disease. Intestingly, using combined lesional and non-lesional samples, we found one or more highly significant DEGs that were involved in epidermal development and morphology. These DEGs did not show up at the top ranking genes when we used either of the AD subtypes. The most important of these was *ARHGAP18*, which showed high FC and low p-value in all of the 5 datasets. This gene has been demonstrated to have a critical role in epidermal cell attachment and polarity [38], but has not yet been described in the context of AD.

The present study also identified major AD functional clusters, gene signatures and enriched pathways relevant to AD classification and etiology as described below.

### 1) Leaky skin barrier and trans-epidermal water-loss related genes were down regulated in AD

Down-regulation of genes expressing epithelial structural proteins (e.g. late cornified envelope *LCE2B*, loricrin, filaggrin, aquaporin and involucrin) have been confirmed. Additionally, we found a down-regulation of the *SCEL* gene which encodes sciellin, a precursor to the cornified envelope of terminally differentiated keratinocytes. Compromised epidermal barrier integrity along with low expression of lipid genes (*FADS1*, *FABP7* and *FAR2* genes) can contribute to trans-epidermal water-loss associated with AD. Down-regulation of lipid genes is very prominent in the case of non-lesional AD. The present study has also identified down-regulation of epidermal growth factors (such as epiregulin) that can disrupt epidermal development. Decreased expression of corneodesmosin (*CDSN*; principal protein in the corneodesmosome structures of human epidermis) and lysyl oxidase (Lox; initiates the crosslinking of collagens and elastin) were observed in all datasets. *CDSN* and Lox, along with other structural proteins (*LCE2B*, *LOR*, *FLG*, and *IVL*) constitute the stratum corneum (SC). Claudin, the principal component protein of the epidermal tight junction, was also significantly down-regulated. This suggests that both stratum corneum and tight junction—two epidermal barrier structures—are defective in AD. Another very interesting candidate was ARHGAP18 (a Rho GTPase activating protein 18) which is involved in cell adherence, spreading and polarity as already mentioned [38, 39].

### 2) Microbial infection—contribution of barrier dysfunction and innate immune genes

Approximately 30% of AD patients suffer from bacterial and viral skin infections and 90% of AD skin lesions are associated with *Staphylococcus* infection [40]. This condition in AD has been linked to a general down-regulation of epidermal innate immune genes (encoding anti-microbial peptides) in contrast to psoriasis, which is usually not associated with significant microbial infections, in spite of the fact that both conditions reflect compromised epidermal barrier function [41, 42]. Enhanced microbial exposure due to a ‘leaky barrier’ can induce the expression of innate immune proteins, such as defensins, that are expressed in negligible levels in normal skin. The diffrentially expressed gene *DEFB4* encodes beta defensin-2, the first human anti-microbial defensin produced upon contact with microorganisms. However, it exhibits activity against gram-negative bacteria (e.g. *E*. *coli*) and *Candida*, but not against gram-positive bacteria (e.g. *S*. *aureus*), which explains why infection caused by gram-negative bacteria are rare in AD. Our analysis did not show any transcription level difference in the expression of beta defensin-3, which kills *S*. *aureus* [43, 44]. However, interestingly, we observed down-regulation of a wide range of innate immune genes such as *MSMB*, *LTF*, *RNASE7* and *SCGB2A1*, which are also responsible for increased susceptibility to microbial infection in AD.

Genes encoding immunoglobulin chains (*IGLV*, *IGLJ*) and *CD1B* (that presents self and non-self lipid and glycolipid antigens to T-cell receptors on natural killer T-cells) show differential expression in response to microbial exposure. Additionally, specific IgE against *Staphylococcal* antigens has been demonstrated in at least one-third of AD patients, which can contribute to allergic inflammation [45]. Enhanced exposure to self-proteins due to itch and compromised barrier function can lead to sensitization to self-proteins [46]. Serum IgE against bacterial /self proteins (unlike in psoriasis) correlate with AD disease severity. AD samples are associated with a marked increase in the S100 family of proteins, particularly S100A7 and S100A8. S100A7 (psoriasin) is a potent chemotactic inflammatory protein for neutrophils and CD4+ T-cells [47]. It is up-regulated in inflammatory skin disorders, while S100A8 and S100A9 form a complex that displays cytostatic and anti-microbial activities [48]. One study comparing gene expression patterns of chronic *versus* acute disease forms showed a sharp up-regulation of S100A7 (psoriasin), S100A8 and S100A9 (calgranulin B) proteins with concomitant development of lesions. Up-regulation of S100A7 and S100A8 in lesions have been demonstrated by immunohistochemistry and quantitative PCR [4]. Up-regulation of S100 proteins has been reported in hyperproliferative epithelium where they were linked to alternative keratinocyte differentiation pathways [49]. Their pro-inflammatory functions as ‘alarmins’ are associated with neutrophil, monocyte and lymphocyte chemotaxis in a number of inflammatory disorders [50]. Antimicrobial functions of S100 family proteins against gram-negative bacteria (such as *E*. *coli*) have also been reported. While AD skin rarely shows *E*. *Coli* infection (in contrast to gram positive *S*. *aureaus* infection), these proteins are probably more related to lymphocyte infiltration in AD lesions [4].

### 3) Inflammation in AD

A consistent upregulation (using 1.5 fold change value) of classic Th2 cytokines such as *IL4* and *IL13* was not observed across datasets. We did notice the upregulation of *IL4* and *IL13* receptor genes in three of the datasets. However, interestingly, the cytokine which was found significantly induced in all of the datasets was *IL1F7* (later named as *IL-37*). This cytokine is a member of IL1 family with suppressor activity on pro-inflammatory cytokine (*IL1A* and *IL6*, *IL23A*) and chemokine (*CCL12*, *CXCL13*) functions. Additionally, it inhibits dendritic cell and IL18 activity. We also noticed an up-regulation of *IL27RA* (receptor for *IL27*) in three out of five datasets. *IL27* is known to involve in clonal expansion of naïve CD4+ cells, stimulation of cytotoxic T-cell activity and down-regulation of inducible regulatory T-cell generation. *CCL18*, the chemokine that showed more than three-fold down-regulation in 4 out of 5 datasets, is related to several disease conditions including AD [51]. The probable source of this chemokine in skin samples are epithelial monocytes, macrophages and DCs [52, 53]. Secreted *CCL18* primarily attracts naïve T-cells (and thus contributes to sensitization) and about 5–10% CLA+ skin-homing T-cells [52–54]. This is consistent with the observation that CLA+CD45RO+ T-cells are the major lymphocyte subtype that infiltrates lesional skin [55, 56]. Furthermore, CCL18 is associated with induction of collagen produced in fibroblasts through the ERK pathway [57], and interestingly, collagen up-regulation was also noted in our AD gene expression datasets. Another gene that contributes to itch, inflammation and dryness in AD is *GREM1* (consistently up-regulated in AD in all of the datasets), which encodes the protein Gremlin. *GREM1* inhibits TGF-beta signaling, thereby promoting inflammation.

### 4) Differentially regulated epidermal proteases and their inhibitors

A potential factor contributing to both barrier dysfunction and inflammation in AD could be dysregulation in protease and protease-inhibitor homeostasis. Genes differentially regulated in this group include serpins (*SERPINB3*, *SERPINB4*), Kallikreins (*KLK5*) and cystatin. Up-regulation of *KLK5* is known to induce AD-like skin lesions in animal models [58]. Moreover, other studies also reported dysregulation of epidermal proteolytic networks in skin disorders [59]. Increased expression of *KLK5* (as seen in AD) augments PAR-2 (protease activated receptor-2) activation, which is directly related to *TSLP* and *IL-8* induction, resulting in epidermal inflammation and pruritus. Induction of AD-like lesions through PAR2-mediated thymic stromal lymphopoietin expression has been demonstrated in Netherton syndrome [58]. *SERPINB3* (squamous cell carcinoma antigen-1) and *SERPINB4* (squamous cell carcinoma antigen-2) were up-regulated in AD. This might be due to secondary microbial protease exposure (e.g. *SERPINB8* and *SERPINB9* are the inhibitors of subtilisin A). Cystatin M is known to be involved in epidermal crosslinking of structural proteins by transglutaminase 3 in the cornification process by controlling cathepsin L and legumain activities [60]. Up-regulation of *KLK5* is known to induce AD-like skin lesions in animal models [58]. Moreover, other studies also reported dysregulation of epidermal proteolytic networks in skin disorders [59]. Uncontrolled cysteine protease activity leads to abnormal stratum corneum and disturbance of skin barrier function [61]. Uncontrolled cysteine protease activity leads to abnormal stratum corneum and disturbance of skin barrier function [61].

### 5) Differentially regulated metabolic enzymes

Several metabolic enzymes produced by the epidermal layer play critical roles in AD pathogenesis. The enzymes showing differential expression in AD include *GALNT6*, *AKR1B10* and *ARHGAP18*. The protein encoded by *GALANT6* is a transferase, which is capable of glycosylating fibronectin peptide, thus taking part in healing and restoration of barrier integrity. Another differentially regulated enzyme *AKR1B10* is a member of Aldo-keto reductase family, which regulates keratinocyte differentiation [62].

### Interplay between major functional gene clusters

Previous studies identified a 1.9Mb region (region 1q21) on chromosome 1 designated as the ‘Epidermal Differentiation Complex (EDC)’ containing several cluster genes including structural genes (*FLG*, *LOR*, *IVL* and *LCE*), *S100A* family genes and S100-fused type genes. These genes encode proteins that have structural and functional similarities to FLG and are abundant in the upper epidermis. Co-localization and strong interconnection between genes encoding epidermal structural proteins and calcium-binding S100 proteins are particularly interesting since calcium level tightly regulate the differentiation of epithelial cells.

Our analysis confirmed a general downregulation of a group of genes related to lipid biosynthesis (Cluster VI). Variants of the differentially regulated gene coding the enzyme fatty acid desaturase (*FADS*), are located on 11q12-q13.1 region. This region has previously been linked to AD [63]. Fatty acid desaturase enzymes catalyze the biosynthesis of highly unsaturated lipids and reduction of epidermal lipid components that have been previously reported. Genes related to epidermal lipid metabolism are directly connected to maintenance of barrier function, prevention of microbial attack and modulating inflammation through downstream processes such as the arachidonic acid pathway [64, 65]. Thus lipid imbalance in keratinocytes can be a major contributing factor for AD pathogenesis.

### Bioinformatic analyses of signature genes suggest biologically relevant networks in AD pathogenesis- potential for bio-medical intervention

Analysis of DEGs using IPA revealed that these 89 signature genes are parts of integrated and interconnected biological networks that play critical roles in AD pathogenesis. (Fig 5A–5C) shows gene networks that connects several genes of the 89ADGES. Network analysis identified *ERK1/2* as an important ‘nodal gene’ connecting several DEGs (Fig 5A). Evidences suggest that this might be involved in histamine-mediated enhanced periostin and collagen production contributing to severity and chronicity of AD [66]. The second network (Fig 5B) indicates the involvement of interferon-gamma signaling pathway in AD. Recently, Ustekinumab,a monoclonal therapeutic antibody directed against interleukin-12 and interleukin-23 (both associated to *IFNG* signaling pathway) has been identified as a potential candidate to treat AD [67]. A number of clinical trials are underway evaluating the efficacy of Ustekinumab to treat AD. Finally, the ‘nodal gene’ *PPARG* (peroxisome proliferator-activated receptor gamma, a ligand-activated nuclear receptors), regulates directly multiple DEGs such as corin, *CCL22* and *S100A8*, and also indirectly regulates several other DEGs (Fig 5C). Interestingly, animal studies suggest that *PPARG* activation directly stimulates profilaggrin expression [68]. Other important ‘nodal gene’ of this network is catenin which is known to confer susceptibility to occupational asthma presumably due to week respiratory epithelial barrier [69]. A critical role of peroxisome proliferator-activated receptors in skin diseases has been emphasized in the literature [70, 71]. An animal model that used combined application of *PPAR* and glucocorticoid has shown promising results [72]. Moreover peroxisome proliferator-activated receptor ligand rosiglitazone has shown promising results in the treatment of AD [73]. However, targeting *PPARA* (peroxisome proliferator-activated receptor alpha) versus *PPARG* for drug development needs be evaluated.

Further analysis using Literature Lab^™^ from Acumenta Biotech showed that the keratinocyte differentiation pathway was significantly (P = 0.0006) enriched in 36 out of the 89 ADGES (Fig 5), many of which are components of the ‘epidermal differentiation complex’ [10, 32–34]. By virtue of their location, keratinocytes provide an important window to the environment and contribute to the development of AD. Keratinocytes are capable of producing several inflammatory mediators. Thus, keratinocytes not only serve a structural factor (as intrinsic defects in barrier function) but also actively participate in inflammatory and immunologic processes [74]. Better understanding of the role of keratinocytes in health and diseases (particularly related to inflammation and defects in lipid homeostatis) might lead to novel topical therapies in the future.

In critically evaluating our results, it is important to note that our analyses, and hence interpretations, are subject to some limitations. First, the present study was not aimed at combining multiple datasets to increase sample size, but to identify genes consistently up/down regulated across multiple datasets. Thus, there was no uniform estimation of expression (preprocessing) of the data, which was normalized within experiment but not across experiments. Therefore, further statistical analyses could be performed to avoid any possible shrinkage/expansion of fold-change values as a result of sample size variations. Second, we analyzed lesional AD and control data across 5 datasets independenly from lesional and non-lesional samples. In future analysis, a mixed modelling approach could be utilized to explore both lesional and non-lesional AD samples against the control as well as paired and unpaired data in the same analysis. Finally, as mentioned above (inflammation in AD), the classical Th2 cytokines *IL4/IL13* were not identified as important DEGS. These genes with very low expression/short half-life typically do not show up in affymetrix chips. These could be further verified using quantitative RT-PCR. However, we found *IL4* gene to be a major nodal gene in lesional AD network analysis.

The present study has also notable strengths. First, we identified major AD functional clusters, gene signatures and enriched keratinocyte pathway relevant to AD classification and etiology. Second, we identified differentially regulated genes across multiple datasets. Functional annotation of these genes implicated their roles in immune responses (e.g., betadefensin, microseminoprotein), keratinocyte differentiation/epidermal development (e.g., *FLG*, *CORIN*, *AQP*, *LOR*, *KRT16*), inflammation (e.g., *IL37*, *IL27RA*, *CCL18*) and lipid metabolism (e.g., *AKR1B10*, *FAD7*, *FAR2*). Third, we identified genes which were not previously described in the context of AD but associated with cell morphology (e.g., *ARHGAP18*). Forth, from network analysis, we identified important nodal genes regulating different functional clusters driving AD pathogenesis (e.g. *PPAR*, *CTNNB1*). Importantly, as already mentioned, PPAR has been recently targeted for AD related drug development.

## Conclusion and Clinical Implication

Using publicly accessible AD gene expression data, we have identified 89 AD signature genes (89ADGES) and the keratinocyte skin barrier pathway as the most enriched pathway in our datasets. Functional annotation of epidermal structural genes indicates that both *stratum corneum* and tight junction barriers of the skin are dysfunctional in AD, associated with increased allergen/irritant exposure, inflammation and microbial infection. This might be due to differential expression of epithelial genes controlling barrier integrity, lipid metabolism, inflammation and innate immunity. Therefore, therapies targeted to increase barrier integrity, rather than just to suppress inflammation, might show more promising results in the treatment of AD. Current management of AD is largely dependent on allergen avoidance, application of moisturizers, corticosteroids, immune-suppressants, with no targeted therapy in clinical use. The AD-associated epidermal protein genes including the role of *FLG* and keratinocyte differentiation pathways identified in this study might be helpful in designing new “barrier therapy” targets for AD. The 89ADGES genes can identify AD patients from healthy individuals with high accuracy, and could also serve as biomarkers for AD therapeutic stratification.

## Supporting Information

S1 Fig Figure shows sample hierarchical clustering (dataset GSE36842) (A) and Principal component analysis of dataset GSE36842 (B). (PPTX)

S2 Fig Figure shows sample volcano plot showing p-value versus fold-change distribution of dataset GSE36842. (PPTX)

S3 Fig qPCR validation of representative up-regulated (KRT6B, SERPINB, CCL22, DEFB4 and S100A8) and down-regulated (CST6, CLDN23 and LOR) genes in a mouse model of atopic dermatitis. Statistical significance between the groups (seven for each) was calculated by the Mann Whitney Test and p-values have been indicated. ASP stands for Aspergillus-treated AD group, while SAL denotes saline-treated control group. This experiment has been performed as a technological confirmation to check the direction of the expression change.
(PPTX)

S1 Table Primer sequences for genes validated using qPCR. Representative upregulated (KRT6B, SERPINB, CCL22, DEFB4 and S100A8) and down-regulated (CST6, CLDN23 and LOR) genes in a mouse model of atopic dermatitis were validated.
(PPTX)

S2 Table Table shows log average fold-change values of 89ADGES with lowest p-value among the five data-sets for up-regulated genes. (PPTX)

S3 Table Table shows log average fold-change values of 89ADGES with lowest p-value among the five data-sets for down-regulated genes. (PPTX)

S4 Table Classification matrix for AD samples based on 89 differentially regulated genes and using discriminant analysis Average accuracy, 98%, n = number of individuals in each study group. (PPTX)
