# Supplementary figures and images for: Multiple Transcriptome Data Analysis Reveals Biologically Relevant Atopic Dermatitis Signature Genes and Pathways
Source: PLoS One. 2015 Dec 30;10(12):e0144316. doi: 10.1371/journal.pone.0144316 (PMC4696650; doi:10.1371/journal.pone.0144316)

## Slide 1
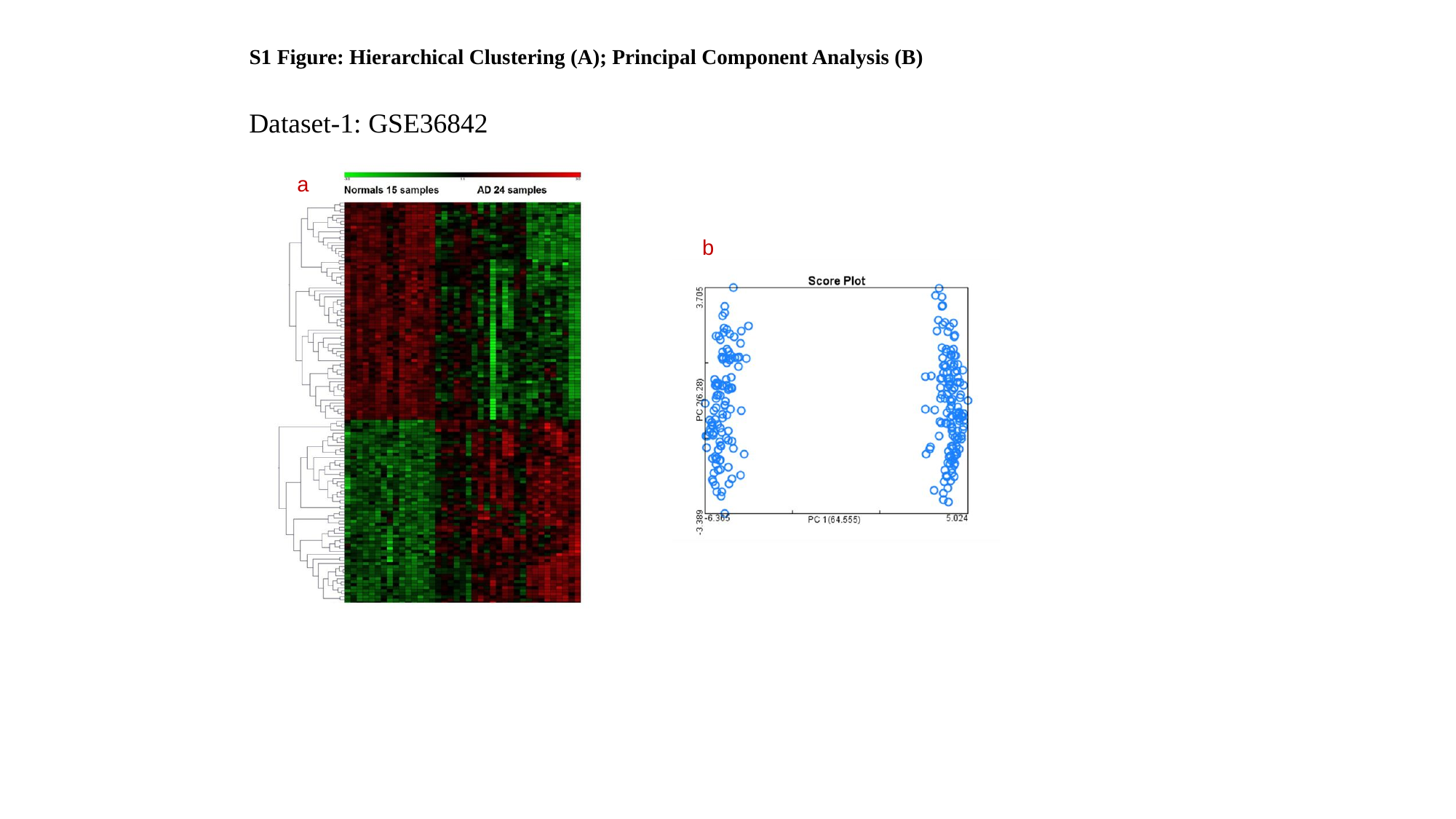

# S1 Figure: Hierarchical Clustering (A); Principal Component Analysis (B)
Dataset-1: GSE36842
a
b

Supplement: S1 Fig — (PPTX) [file pone.0144316.s001.pptx]
