# Supplementary material for: Multiple Transcriptome Data Analysis Reveals Biologically Relevant Atopic Dermatitis Signature Genes and Pathways
Source: PLoS One. 2015 Dec 30;10(12):e0144316. doi: 10.1371/journal.pone.0144316 (PMC4696650; doi:10.1371/journal.pone.0144316)
Supplement: S2 Fig — (PPTX) [file pone.0144316.s002.pptx]

## Slide 1
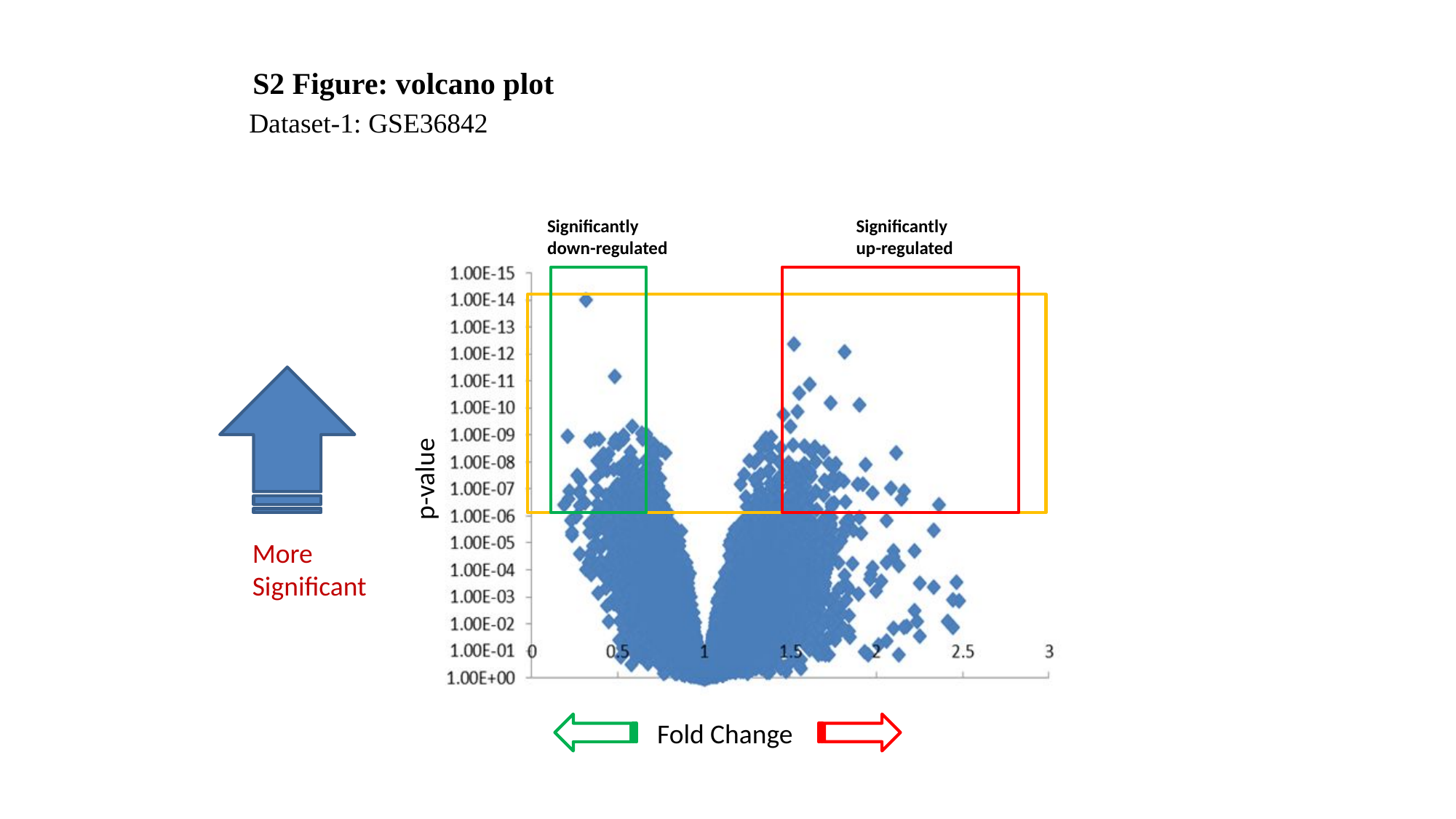

# S2 Figure: volcano plot
Dataset-1: GSE36842
Significantly
up-regulated
Significantly
down-regulated
p-value
More
Significant
Fold Change
