# Supplementary material for: Multiple Transcriptome Data Analysis Reveals Biologically Relevant Atopic Dermatitis Signature Genes and Pathways
Source: PLoS One. 2015 Dec 30;10(12):e0144316. doi: 10.1371/journal.pone.0144316 (PMC4696650; doi:10.1371/journal.pone.0144316)
Supplement: S3 Fig — Statistical significance between the groups (seven for each) was calculated by the Mann Whitney Test and p-values have been indicated. ASP stands for Aspergillus-treated AD group, while SAL denotes saline-treated control group. This experiment has been performed as a technological confirmation to check the direction of the expression change. (PPTX) [file pone.0144316.s003.pptx]

## Slide 1
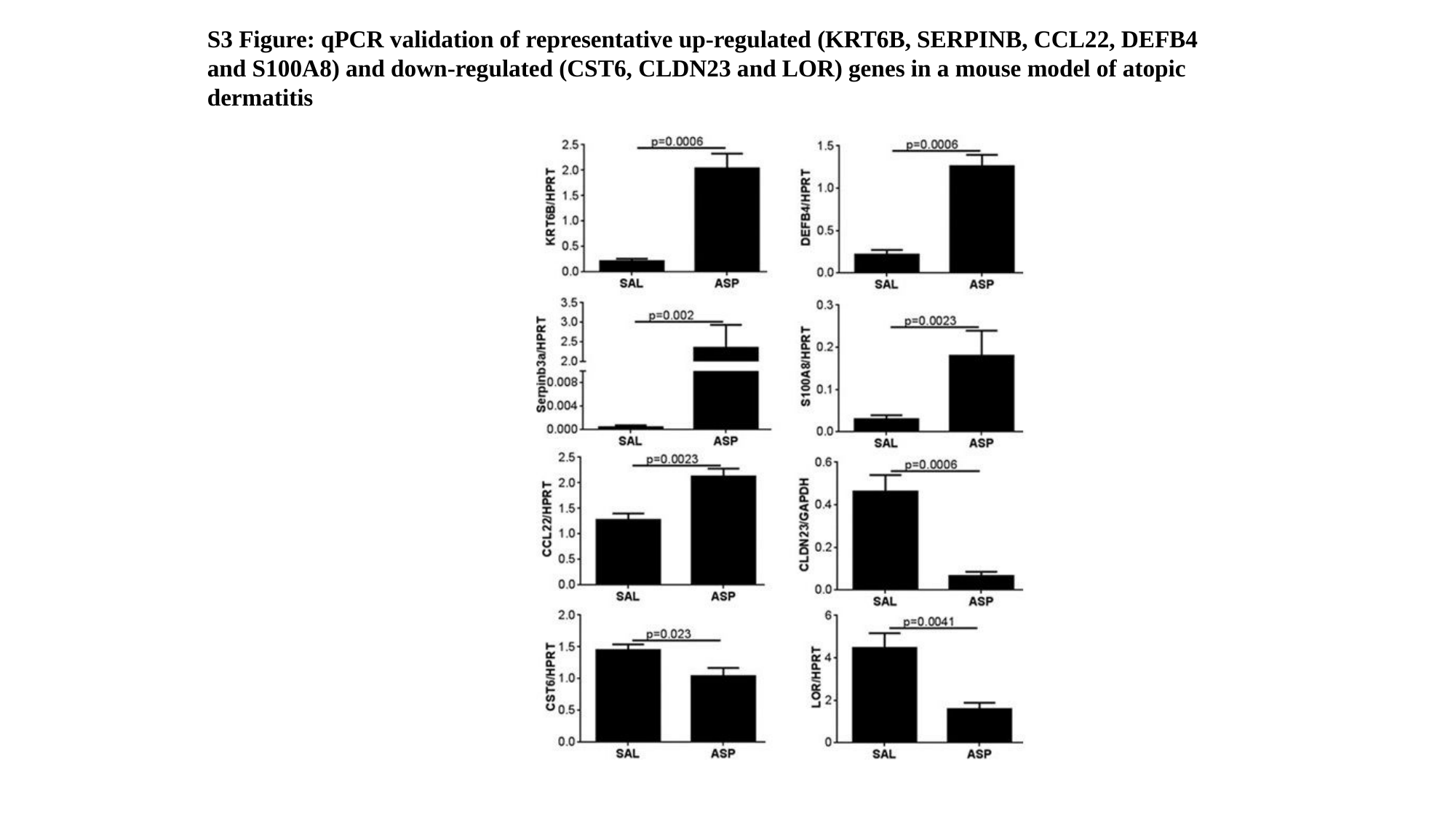

S3 Figure: qPCR validation of representative up-regulated (KRT6B, SERPINB, CCL22, DEFB4 and S100A8) and down-regulated (CST6, CLDN23 and LOR) genes in a mouse model of atopic dermatitis
