# Supplementary material for: Multiple Transcriptome Data Analysis Reveals Biologically Relevant Atopic Dermatitis Signature Genes and Pathways
Source: PLoS One. 2015 Dec 30;10(12):e0144316. doi: 10.1371/journal.pone.0144316 (PMC4696650; doi:10.1371/journal.pone.0144316)
Supplement: S1 Table — Representative upregulated (KRT6B, SERPINB, CCL22, DEFB4 and S100A8) and down-regulated (CST6, CLDN23 and LOR) genes in a mouse model of atopic dermatitis were validated. (PPTX) [file pone.0144316.s004.pptx]

## Slide 1
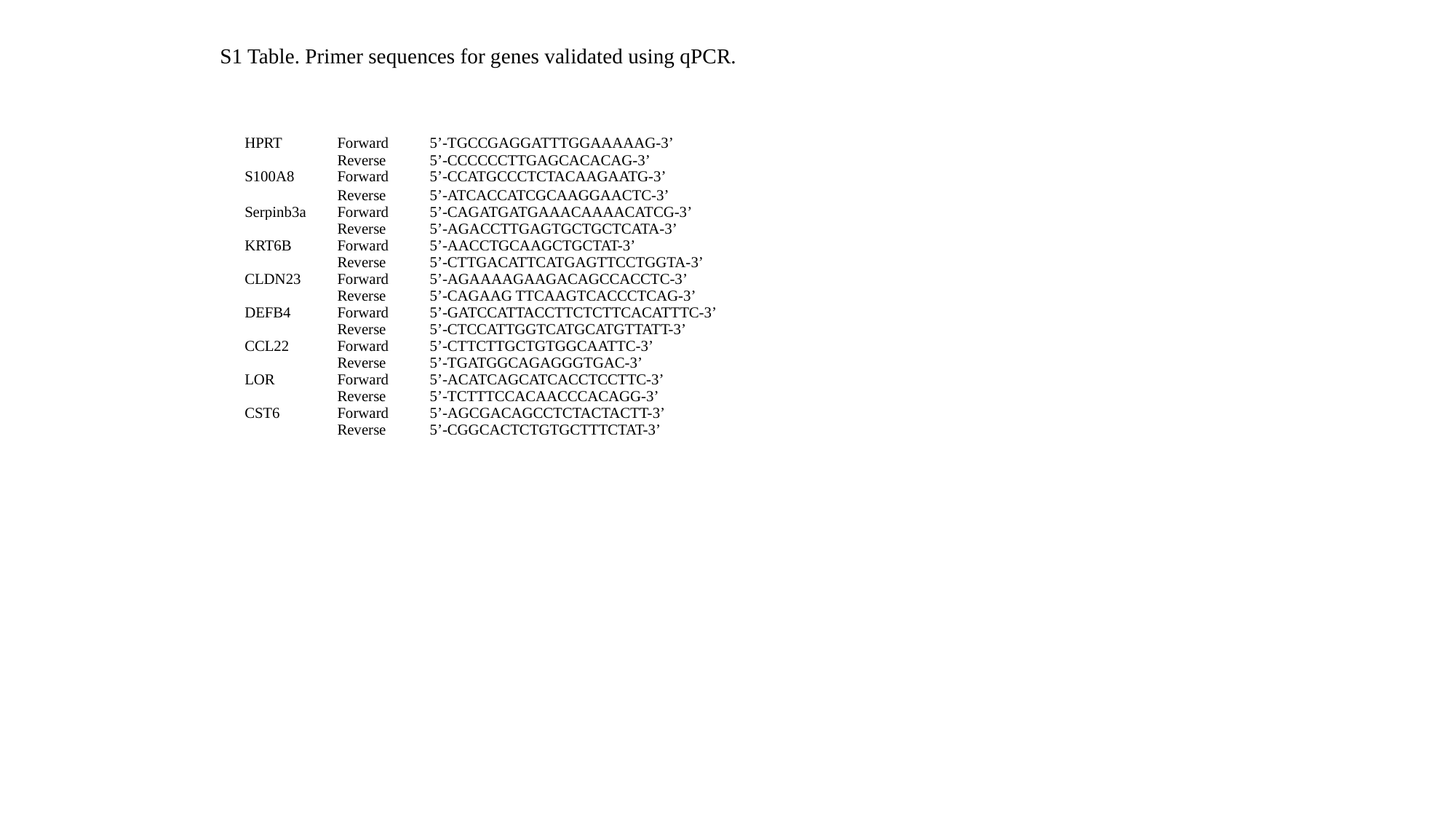

S1 Table. Primer sequences for genes validated using qPCR.
| | | Primer sequences |
| --- | --- | --- |
| HPRT | Forward | 5’-TGCCGAGGATTTGGAAAAAG-3’ |
| | Reverse | 5’-CCCCCCTTGAGCACACAG-3’ |
| S100A8 | Forward | 5’-CCATGCCCTCTACAAGAATG-3’ |
| | Reverse | 5’-ATCACCATCGCAAGGAACTC-3’ |
| Serpinb3a | Forward | 5’-CAGATGATGAAACAAAACATCG-3’ |
| | Reverse | 5’-AGACCTTGAGTGCTGCTCATA-3’ |
| KRT6B | Forward | 5’-AACCTGCAAGCTGCTAT-3’ |
| | Reverse | 5’-CTTGACATTCATGAGTTCCTGGTA-3’ |
| CLDN23 | Forward | 5’-AGAAAAGAAGACAGCCACCTC-3’ |
| | Reverse | 5’-CAGAAG TTCAAGTCACCCTCAG-3’ |
| DEFB4 | Forward | 5’-GATCCATTACCTTCTCTTCACATTTC-3’ |
| | Reverse | 5’-CTCCATTGGTCATGCATGTTATT-3’ |
| CCL22 | Forward | 5’-CTTCTTGCTGTGGCAATTC-3’ |
| | Reverse | 5’-TGATGGCAGAGGGTGAC-3’ |
| LOR | Forward | 5’-ACATCAGCATCACCTCCTTC-3’ |
| | Reverse | 5’-TCTTTCCACAACCCACAGG-3’ |
| CST6 | Forward | 5’-AGCGACAGCCTCTACTACTT-3’ |
| | Reverse | 5’-CGGCACTCTGTGCTTTCTAT-3’ |
