# Supplementary material for: Multiple Transcriptome Data Analysis Reveals Biologically Relevant Atopic Dermatitis Signature Genes and Pathways
Source: PLoS One. 2015 Dec 30;10(12):e0144316. doi: 10.1371/journal.pone.0144316 (PMC4696650; doi:10.1371/journal.pone.0144316)
Supplement: S2 Table — (PPTX) [file pone.0144316.s005.pptx]

## Slide 1
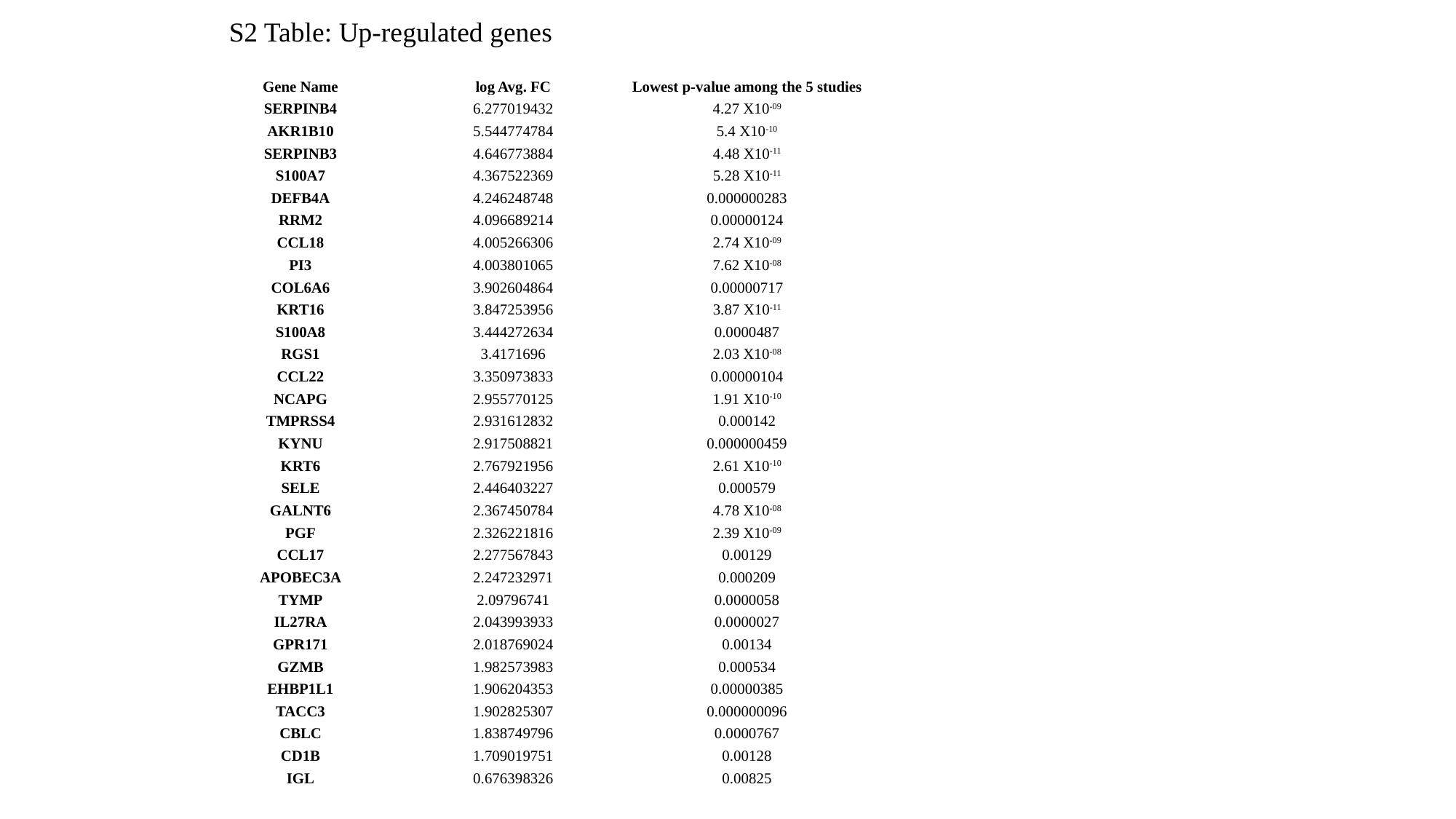

S2 Table: Up-regulated genes
| Gene Name | log Avg. FC | Lowest p-value among the 5 studies |
| --- | --- | --- |
| SERPINB4 | 6.277019432 | 4.27 X10-09 |
| AKR1B10 | 5.544774784 | 5.4 X10-10 |
| SERPINB3 | 4.646773884 | 4.48 X10-11 |
| S100A7 | 4.367522369 | 5.28 X10-11 |
| DEFB4A | 4.246248748 | 0.000000283 |
| RRM2 | 4.096689214 | 0.00000124 |
| CCL18 | 4.005266306 | 2.74 X10-09 |
| PI3 | 4.003801065 | 7.62 X10-08 |
| COL6A6 | 3.902604864 | 0.00000717 |
| KRT16 | 3.847253956 | 3.87 X10-11 |
| S100A8 | 3.444272634 | 0.0000487 |
| RGS1 | 3.4171696 | 2.03 X10-08 |
| CCL22 | 3.350973833 | 0.00000104 |
| NCAPG | 2.955770125 | 1.91 X10-10 |
| TMPRSS4 | 2.931612832 | 0.000142 |
| KYNU | 2.917508821 | 0.000000459 |
| KRT6 | 2.767921956 | 2.61 X10-10 |
| SELE | 2.446403227 | 0.000579 |
| GALNT6 | 2.367450784 | 4.78 X10-08 |
| PGF | 2.326221816 | 2.39 X10-09 |
| CCL17 | 2.277567843 | 0.00129 |
| APOBEC3A | 2.247232971 | 0.000209 |
| TYMP | 2.09796741 | 0.0000058 |
| IL27RA | 2.043993933 | 0.0000027 |
| GPR171 | 2.018769024 | 0.00134 |
| GZMB | 1.982573983 | 0.000534 |
| EHBP1L1 | 1.906204353 | 0.00000385 |
| TACC3 | 1.902825307 | 0.000000096 |
| CBLC | 1.838749796 | 0.0000767 |
| CD1B | 1.709019751 | 0.00128 |
| IGL | 0.676398326 | 0.00825 |
