# Supplementary material for: Multiple Transcriptome Data Analysis Reveals Biologically Relevant Atopic Dermatitis Signature Genes and Pathways
Source: PLoS One. 2015 Dec 30;10(12):e0144316. doi: 10.1371/journal.pone.0144316 (PMC4696650; doi:10.1371/journal.pone.0144316)
Supplement: S3 Table — (PPTX) [file pone.0144316.s006.pptx]

## Slide 1
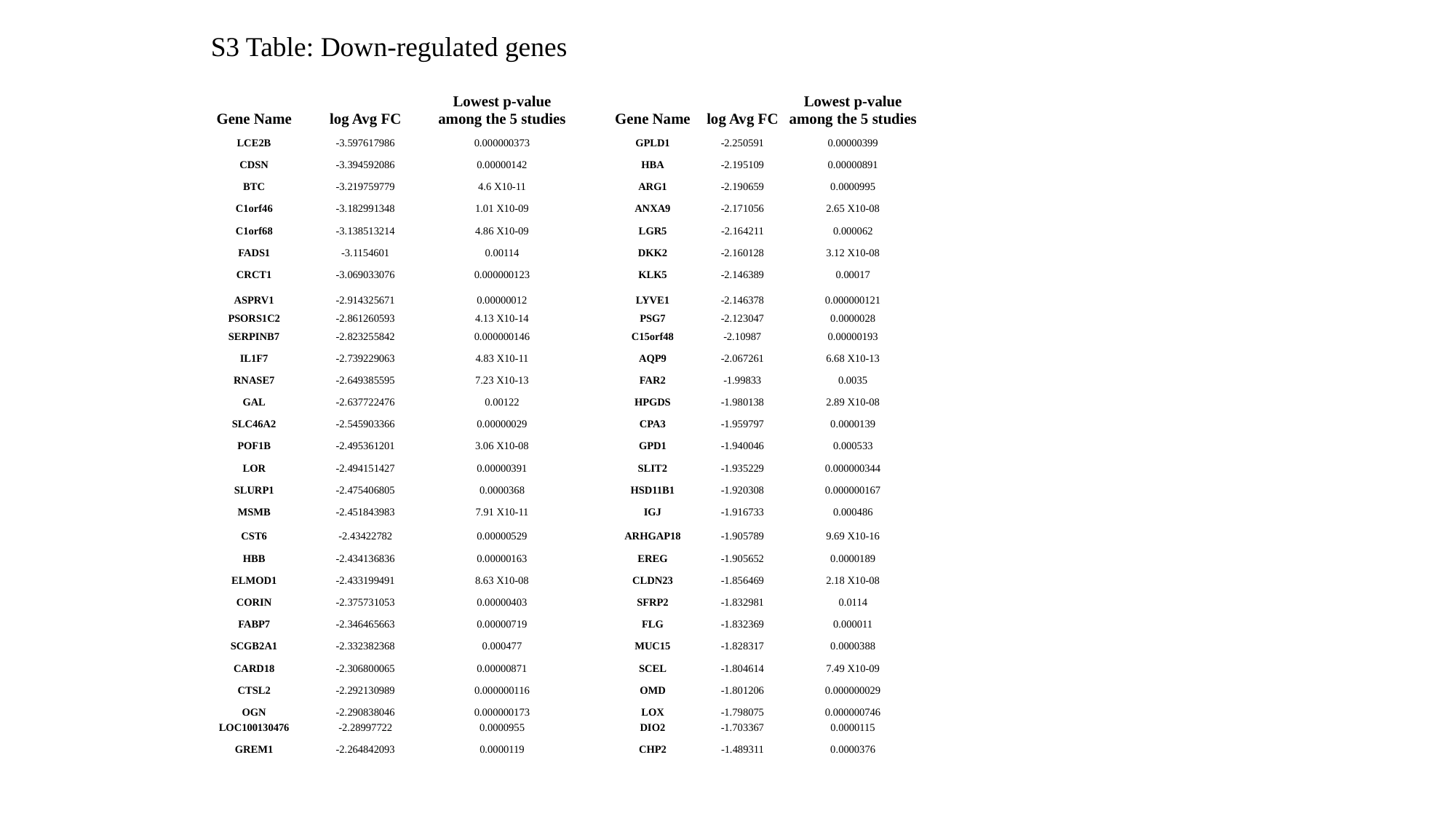

S3 Table: Down-regulated genes
| Gene Name | log Avg FC | Lowest p-value among the 5 studies | | Gene Name | log Avg FC | Lowest p-value among the 5 studies |
| --- | --- | --- | --- | --- | --- | --- |
| LCE2B | -3.597617986 | 0.000000373 | | GPLD1 | -2.250591 | 0.00000399 |
| CDSN | -3.394592086 | 0.00000142 | | HBA | -2.195109 | 0.00000891 |
| BTC | -3.219759779 | 4.6 X10-11 | | ARG1 | -2.190659 | 0.0000995 |
| C1orf46 | -3.182991348 | 1.01 X10-09 | | ANXA9 | -2.171056 | 2.65 X10-08 |
| C1orf68 | -3.138513214 | 4.86 X10-09 | | LGR5 | -2.164211 | 0.000062 |
| FADS1 | -3.1154601 | 0.00114 | | DKK2 | -2.160128 | 3.12 X10-08 |
| CRCT1 | -3.069033076 | 0.000000123 | | KLK5 | -2.146389 | 0.00017 |
| ASPRV1 | -2.914325671 | 0.00000012 | | LYVE1 | -2.146378 | 0.000000121 |
| PSORS1C2 | -2.861260593 | 4.13 X10-14 | | PSG7 | -2.123047 | 0.0000028 |
| SERPINB7 | -2.823255842 | 0.000000146 | | C15orf48 | -2.10987 | 0.00000193 |
| IL1F7 | -2.739229063 | 4.83 X10-11 | | AQP9 | -2.067261 | 6.68 X10-13 |
| RNASE7 | -2.649385595 | 7.23 X10-13 | | FAR2 | -1.99833 | 0.0035 |
| GAL | -2.637722476 | 0.00122 | | HPGDS | -1.980138 | 2.89 X10-08 |
| SLC46A2 | -2.545903366 | 0.00000029 | | CPA3 | -1.959797 | 0.0000139 |
| POF1B | -2.495361201 | 3.06 X10-08 | | GPD1 | -1.940046 | 0.000533 |
| LOR | -2.494151427 | 0.00000391 | | SLIT2 | -1.935229 | 0.000000344 |
| SLURP1 | -2.475406805 | 0.0000368 | | HSD11B1 | -1.920308 | 0.000000167 |
| MSMB | -2.451843983 | 7.91 X10-11 | | IGJ | -1.916733 | 0.000486 |
| CST6 | -2.43422782 | 0.00000529 | | ARHGAP18 | -1.905789 | 9.69 X10-16 |
| HBB | -2.434136836 | 0.00000163 | | EREG | -1.905652 | 0.0000189 |
| ELMOD1 | -2.433199491 | 8.63 X10-08 | | CLDN23 | -1.856469 | 2.18 X10-08 |
| CORIN | -2.375731053 | 0.00000403 | | SFRP2 | -1.832981 | 0.0114 |
| FABP7 | -2.346465663 | 0.00000719 | | FLG | -1.832369 | 0.000011 |
| SCGB2A1 | -2.332382368 | 0.000477 | | MUC15 | -1.828317 | 0.0000388 |
| CARD18 | -2.306800065 | 0.00000871 | | SCEL | -1.804614 | 7.49 X10-09 |
| CTSL2 | -2.292130989 | 0.000000116 | | OMD | -1.801206 | 0.000000029 |
| OGN | -2.290838046 | 0.000000173 | | LOX | -1.798075 | 0.000000746 |
| LOC100130476 | -2.28997722 | 0.0000955 | | DIO2 | -1.703367 | 0.0000115 |
| GREM1 | -2.264842093 | 0.0000119 | | CHP2 | -1.489311 | 0.0000376 |
