# Supplementary material for: Multiple Transcriptome Data Analysis Reveals Biologically Relevant Atopic Dermatitis Signature Genes and Pathways
Source: PLoS One. 2015 Dec 30;10(12):e0144316. doi: 10.1371/journal.pone.0144316 (PMC4696650; doi:10.1371/journal.pone.0144316)
Supplement: S4 Table — (PPTX) [file pone.0144316.s007.pptx]

## Slide 1
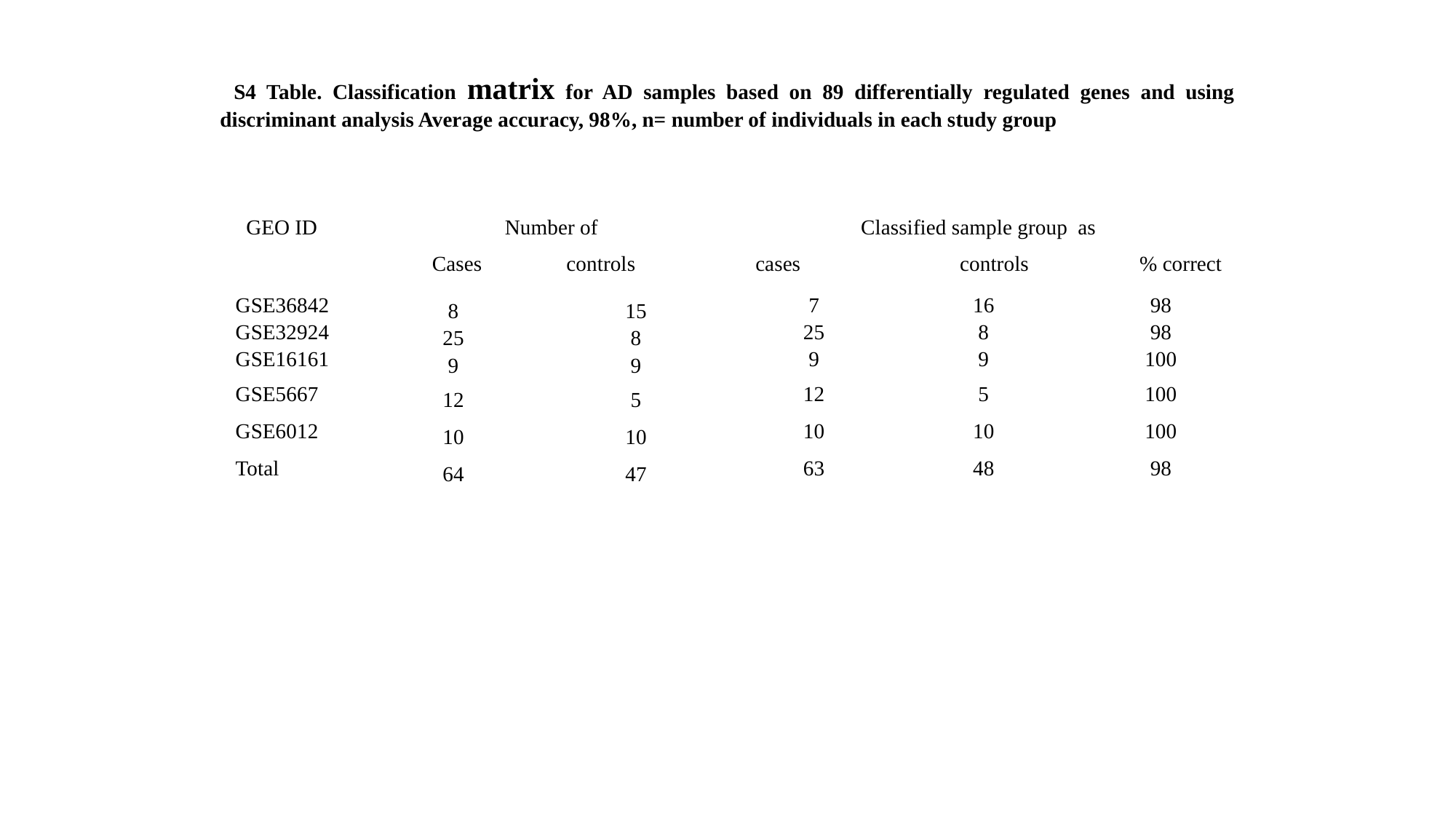

S4 Table. Classification matrix for AD samples based on 89 differentially regulated genes and using discriminant analysis Average accuracy, 98%, n= number of individuals in each study group
| GEO ID | Number of Cases controls | | Classified sample group as   cases controls % correct | | |
| --- | --- | --- | --- | --- | --- |
| GSE36842 | 8 | 15 | 7 | 16 | 98 |
| GSE32924 | 25 | 8 | 25 | 8 | 98 |
| GSE16161 | 9 | 9 | 9 | 9 | 100 |
| GSE5667 | 12 | 5 | 12 | 5 | 100 |
| GSE6012 | 10 | 10 | 10 | 10 | 100 |
| Total | 64 | 47 | 63 | 48 | 98 |
